# Plasma Ang2 and ADAM17 levels are elevated during clinical malaria; Ang2 level correlates with severity and expression of EPCR-binding PfEMP1

**DOI:** 10.1038/srep35950

**Published:** 2016-10-27

**Authors:** Jens E. V. Petersen, Sixbert I. Mkumbaye, Anna V. Vaaben, Alphaxard Manjurano, Eric Lyimo, Reginald A. Kavishe, Steven B. Mwakalinga, Jacklin Mosha, Daniel T. R. Minja, John P. A. Lusingu, Thor G. Theander, Thomas Lavstsen, Christian W. Wang

**Affiliations:** 1 Centre for Medical Parasitology, Department of Immunology & Microbiology, Faculty of Health and Medical Sciences, University of Copenhagen and Department of Infectious Diseases, Rigshospitalet, Copenhagen, Denmark; 2 Kilimanjaro Christian Medical University College and Kilimanjaro Clinical Research Institute, Moshi, Tanzania; 3 National Institute for Medical Research, Mwanza Research Centre, Mwanza, Tanzania; 4 National Institute for Medical Research, Tanga Centre, Tanga, Tanzania

## Abstract

The pathogenesis of *Plasmodium falciparum* malaria involves a complex interplay between parasite adhesion and inflammatory response that includes release of cytokines and activation of the endothelium with accompanying release of Angiopoitin 2 (Ang2) to the plasma. A-disintegrin and metalloproteinase 17 (ADAM17) is a protein responsible for releasing cytokines, including Tumor Necrosis Factor α (TNFα), and shedding of adhesion proteins. In this study, we show that plasma levels of ADAM17 are increased in Tanzanian children hospitalized with a malaria infection compared with asymptomatic children but similar to children hospitalized with other infectious diseases. The plasma levels of ADAM17 decreased during recovery after an acute malaria episode. Plasma levels of Ang2 were associated with markers of malaria severity and levels of *var* transcripts encoding *P. falciparum* Erythrocyte Membrane Protein 1 (PfEMP1) containing Cysteine Rich Inter Domain Region α1 (CIDRα1) domains predicted to bind Endothelial Protein C receptor (EPCR). ADAM17 levels were not associated with expression of *var* genes encoding different PfEMP1 types when controlling for age. These data are the first to report ADAM17 plasma levels in malaria-exposed individuals, and support the notion that parasite sequestration mediated by EPCR-binding PfEMP1 is associated with endothelial activation and pathology in severe paediatric malaria.

In 2015, there were between 150 and 300 million malaria cases worldwide, causing an estimated 438,000 deaths of which 90% occurred in Sub-Saharan Africa^1^. The most vulnerable group is children under the age of five infected with *Plasmodium falciparum.* Even with the current artemisinin combination treatments, infected individuals are at a high risk of dying if they develop symptoms or signs of severe malaria (SM). Clinical manifestations of SM are typically cerebral malaria (CM), respiratory distress (RD), severe malarial anaemia (SMA), and hyperparasitaemia and they often overlap in individual patients. A hallmark of a *P. falciparum* infection is the sequestration of infected erythrocytes (IEs) to the host microvasculature^2^. To avoid splenic clearance, the parasite adheres to the vasculature by expressing *P. falciparum* Erythrocyte Membrane Protein 1 (PfEMP1) binding infected erthrocytes to endothelial host receptors such as cluster of differentiation 36 (CD36) and endothelial protein C receptor (EPCR)^3^.

PfEMP1s are encoded by *var* genes, and each *P. falciparum* genome contains about 60 different *var* genes that are divided into four main groups: A, B, C, and *var2csa,* correlating both with genomic location and direction of transcription, sequence similarity and receptor binding phenotype^3^,^4^,^5^,^6^. Thus, group A PfEMP1s, characterized by having an N-terminal Duffy-binding like α1 (DBLα1) domains followed by either a CIDRα1 domain, which is predicted to bind EPCR or a CIDRβ, γ, or δ domain, which are potentially linked to rosetting (IE binding of uninfected erythrocytes). Group B and C PfEMP1s have N-terminal DBLα0-CIDRα2-6 domain cassettes (co-occurring domain subtypes) predicted to bind CD36. A special set of PfEMP1s, the domain cassette 8 (DC8) PfEMP1, have evolved as an intermediate of group B and A PfEMP1. The DC8 PfEMP1 also bind EPCR via the CIDRα1.1 or 1.8 domain subtypes. Parasite sequestration is a major driver of the pathogenesis of an *P. falciparum* infection^2^, and sequestration via EPCR-binding PfEMP1s has been strongly associated with the development of SM^3^,^7^,^8^,^9^.

*P. falciparum* infections activate the innate immune system causing release of inflammatory cytokines. Of particular importance is Tumor Necrosis Factor α (TNFα), a proinflammatory cytokine that induces fever, cachexia, and apoptosis of endothelial cells^10^,^11^. TNFα is increased in malaria patients and has been associated with the severity of infection^12^. TNFα is released by A-disintegrin and metalloproteinase 17 (ADAM17), primarily from macrophages where it is expressed on the surface and subsequently cleaved off^13^. ADAM17 was initially discovered as the metalloproteinase responsible for cleaving and releasing TNFα and is therefore also known as TNFα converting enzyme (TACE)^13^.

ADAM17 mediates shedding of multiple other cytokines and cytokine receptors, including colony stimulating factor 1 (CSF1), TNFα receptor (TNFαR) and interleukin 6 receptor (IL6R)^14^. ADAM17 also mediates shedding of proteins involved in leukocyte-endothelial cell interactions, such as L-selectin and intercellular adhesion molecule-1 (ICAM-1)^14^,^15^, as well as a range of other surface receptors including EPCR^14^,^16^. Thus, ADAM17 may influence availability of host receptors utilized by IE in malaria. Absence of EPCR-staining at sites of the brain with sequestering IEs suggests that EPCR is cleaved off the endothelium or internalized as a consequence of sequestration, potentially exacerbating the vascular pathology by impairing the functions of activated Protein C (APC)^17^.

The interaction between EPCR and PfEMP1 is also thought to enhance the vascular pathology, as PfEMP1 binding to EPCR blocks the binding of the natural EPCR ligand, Protein C (PC), *in vitro*^6^,^18^. *In vitro,* PfEMP1 binding to EPCR reduces PC activation and dampens APC’s anti-inflammatory effects, including the ability to protect against thrombin-induced barrier disruption^19^,^20^. In the absence of APC-EPCR engagement, thrombin cleaving of protease-activated receptor 1 (PAR1) results in enhanced signaling for release of Weibel-Palade Bodies (WPBs).

Angiopoietin 2 (Ang2) is a growth factor molecule released from WPBs^21^. Elevated plasma concentrations of Ang2 reflect endothelial activation and are correlated with SM and risk of death^22^,^23^. Ang2 competes with Ang1 for binding to the receptor tyrosine kinase Tie2, which upon Ang2 binding decreases endothelial barrier integrity. The auto-/paracrine signaling initiated by Ang2 is involved in the transmigration of leukocytes by destabilizing the endothelium^24^,^25^.

In addition to the presence on leukocytes, ADAM17 is also found along with Ang2 in the WPBs of endothelial cells^26^. Little is known about ADAM17 in malaria, but the cleavage of host receptors specifically utilized by IEs in severe malaria patients and cytokines, such as TNFα, which are increased in SM, suggests ADAM17 plays a role in malaria pathogenesis. This study investigated the plasma levels of ADAM17 and Ang2 in Tanzanian children admitted to the hospital with UM, SM, or with other infections, and the potential correlations between ADAM17 or Ang2 and PfEMP1 subtype expression.

## Results

### Study population

Characteristics of the study population are presented in Table 1. Children with CM, SMA, RD, hyperparasitaemia were diagnosed as severe malaria (SM). The patients in UM and SM groups were of equal age distributions, the children hospitalized with infections other than malaria were of younger age (P < 0.0001, Table 1), while the children with asymptomatic malaria infections were older (P = 0.02, Table 1).

### Plasma ADAM17 is elevated in hospitalized children

**Figure 1:**
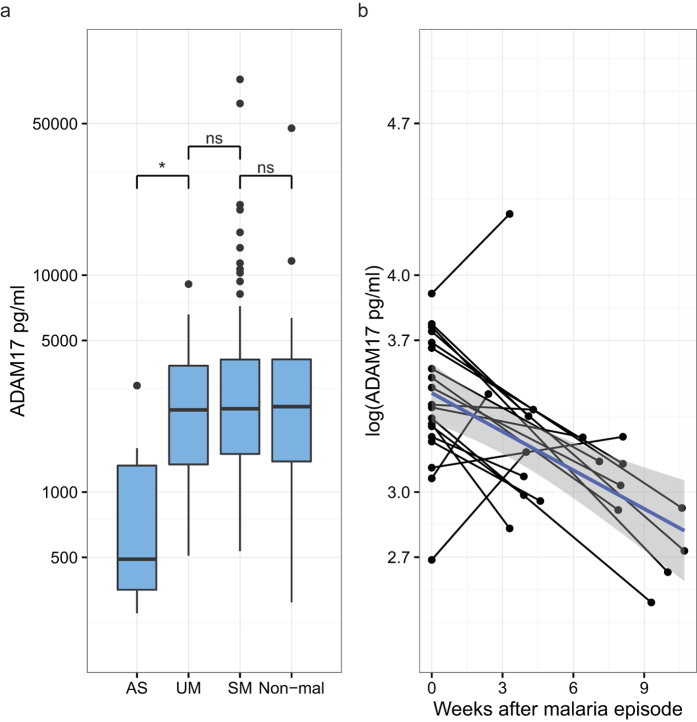
ADAM17 plasma levels. (**a**) Boxplot of ADAM17 plasma levels for children with either asymptomatic malaria (AS, n = 6), uncomplicated malaria (UM, n = 39), severe malaria (SM, n = 123), or children admitted to the hospital without malaria (Non-mal, n = 32). The boxplots display median, 1^st^ quartile (Q1), and 3^rd^ quartile (Q3). The lower whisker indicates the lowest data point above Q1 minus 1.5 times IQR, the upper whisker indicates the highest data point below 1.5 IQR plus Q3. Values below Q1 minus 1.5 IQR and above Q3 plus 1.5 IQR are indicated as points. Statistical significance was calculated using Kruskal-Wallis test and pairwise comparisons using Dunn’s Test with Bonferroni correction. *P < 0.05. (**b**) Acute/convalescent plasma levels of ADAM17 samples from 19 children hospitalized with malaria. Acute samples taken at t = 0, convalescent follow-up samples were taken between 2 and 12 weeks after the hospitalization. ADAM17 data is log-transformed. Linear model in blue with 95% confidence interval in grey (P-value = 0.0001).

To determine whether ADAM17 plasma levels were associated with clinical malaria, we measured ADAM17 plasma levels by ELISA. Children with UM had a median ADAM17 plasma level of 2,329 pg/mL, and children with SM had a similar median of 2,423 pg/mL (Fig. 1a). Asymptomatic children had lower levels of ADAM17 than children with UM or SM with a median of 490 pg/ml (P = 0.04 and P = 0.01, respectively). The median ADAM17 level for the non-malaria patient group was 2,418 pg/ml, similar to UM and SM patients. We observed no difference in the ADAM17 levels between UM and SM patients of Korogwe and Magu. The non-malaria patient group had a higher median level than the asymptomatic children (P = 0.03). Plasma levels of ADAM17 during acute malaria were compared to plasma levels during recovery in samples collected at admission from hospitalized children (Table 1) and from the same children between 2 to 12 weeks after admission (Fig. 1b). In the plasma from convalescent patients, the ADAM17 levels were significantly decreased to a median of 1,180 pg/ml (P < 0.0001). There was no statistically significant difference between ADAM17 levels in convalescent patients and the asymptomatic malaria children (P = 0.2), and ADAM17 plasma levels samples were negatively correlated with the time progressed since diagnosis (Fig. 1b, P = 0.0001). Among non-malaria patients, the ADAM17 levels did not differ significantly between the following three groups; septicemia and/or meningitis, pneumonia or submandibular swelling, and those with gastroenteritis or urinary tract infections (P = 0.3, Fig. S1a). ADAM17 levels were also measured in the plasma from 33 healthy Danish adults (median of 377 pg/ml) and were similar to the levels found in the asymptomatic children (Supplementary Fig. S2a).

### Ang2 plasma levels are elevated in children with severe malaria

**Figure 2:**
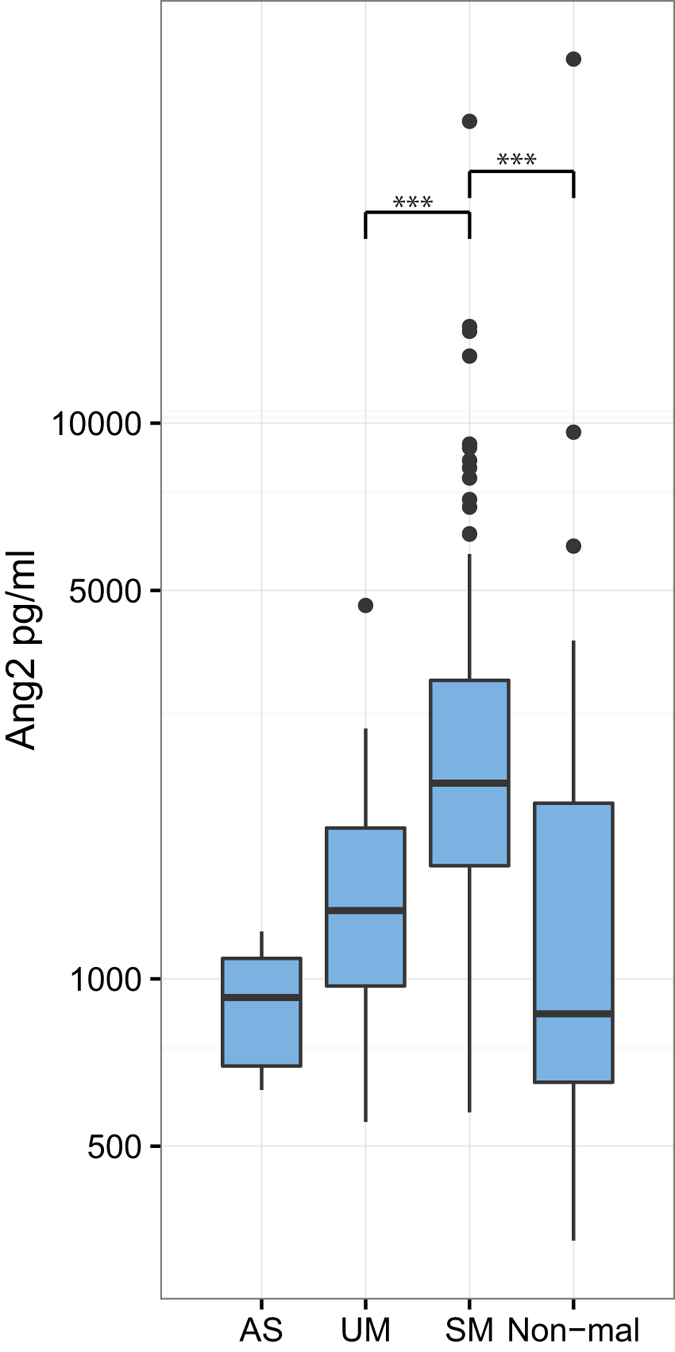
Angiopoietin 2 (Ang2) plasma levels. Boxplot of Ang2 plasma levels for children with either asymptomatic malaria (AS, n = 6), uncomplicated malaria (UM, n = 39), severe malaria (SM, n = 123), or children admitted to the hospital without malaria (Non-mal, n = 32 The boxplots display median, 1^st^ quartile (Q1), and 3^rd^ quartile (Q3). The lower whisker indicates the lowest data point above Q1 minus 1.5 times IQR, the upper whisker indicates the highest datapoint below 1.5 IQR plus Q3. Values below Q1 minus 1.5 IQR and above Q3 plus 1.5 IQR are indicated as points. Statistical significance was calculated using Kruskal-Wallis test and pairwise comparisons using Dunn’s Test with Bonferroni correction. ***P < 0.0001.

Ang2 plasma levels have previously been shown to correlate with the severity of a malaria infection^23^. We found that Ang2 levels were significantly higher in SM patients compared with UM patients (SM median 2,195 pg/ml vs. UM median 1,461 pg/ml, P < 0.0001, Fig. 2). The median Ang2 plasma levels in individuals with asymptomatic malaria (median 926 pg/ml) or hospitalized with non-malaria infections (median 884 pg/ml) were both significantly lower than in the SM group (P < 0.0001). There was no statistically significant difference between the Ang2 levels of those with UM and asymptomatic malaria. Within the non-malaria groups, the Ang2 levels did not differ significantly between the different non-malaria cases (P = 0.4, Supplementary Fig. S1b). In plasma from healthy Danish adults we found a median Ang2 level of 576 pg/ml (Supplementary Fig. S2b). When looking at Magu and Korogwe individually, both had an increased Ang2 in SM compared to UM (P = 0.0002 and P = 0.0004, respectively). The levels of Ang2 in SM patients were higher in Magu compared with Korogwe (P < 0.0001), while the levels of Ang2 in UM patients did not differ significantly (P = 0.1).

### Ang2 but not ADAM17 plasma levels are associated with malaria severity in hospitalized children

As we found no difference in ADAM17 levels between the UM and SM groups, these were grouped for the further analysis of factors or variables correlated with the ADAM17 levels in patients hospitalized with malaria. We found no correlation with any of the symptoms or signs previously associated with increased risk of fatal outcome of a malaria infection and plasma ADAM17 levels, such as low Hb, high lactate, or Blantyre coma score <3. ADAM17 levels were correlated with Ang2 (P = 0.002, Table 2), weakly correlated with the marker for parasite biomass HRP2 (although not statistically significant when correcting for multiple testing, Rho = 0.17, P = 0.03), and ADAM17 levels were inversely correlated with age (P = 0.001). By contrast, Ang2 plasma levels were inversely correlated with Blantyre coma score (P = 0.0002) and Hb-levels (P < 0.0001) and positively correlated with lactate (P < 0.0001), but had no significant correlation with age or parasitaemia (both when measured as circulating erythrocytes or by analyzing plasma levels of HRP2) (Table 2).

### Ang2 but not ADAM17 plasma levels are associated with PfEMP1 type expression

IE sequestration via the PfEMP1-EPCR interaction is thought to affect pathology through occlusion of blood flow and promotion of coagulation and inflammation^27^.

We utilized *var* transcript profiles of the UM and SM individuals (Mkumbaye *et al*., submitted), to explore Ang2 and ADAM17 plasma level correlations with expression of specific *var* subsets, encoding PfEMP1 predicted to bind or not to bind EPCR. Group A PfEMP1 expression levels were estimated by the “DBLa1all” primer set. The DBLa1all reported expression levels that correlated with Ang2 plasma levels but not with ADAM17 plasma levels (Table 2). Group A transcripts targeted by the DBLa2/1.1/2/4/7 primers typically encode EPCR-binding CIDRα1 domains^7^. Abundance of such transcripts also weakly correlated with Ang2 (Rho = 0.25, P = 0.002), whereas group A transcripts encoding PfEMP1 *not* binding EPCR (reported by the DBLa1.5/6/8 primers) were not correlated with Ang2 plasma levels (P = 0.27) (Table 2). When investigating transcript levels reported by primers specific for genes encoding different EPCR-binding CIDRα1 subtypes, we found a weak correlation between plasma levels of Ang2 and the abundance of transcripts encoding CIDRα1.1 (DC8) (Rho = 0.25, P = 0.001, Table 2) and CIDRα1.4 (DC13) domains, although CIDRα1.4 was not statistically significant when using Bonferroni’s correction for multiple testing (Rho = 0.18, P = 0.02; Table 2). ADAM17 plasma levels were weakly correlated with the abundance of CIDRα1.1 encoding transcripts, however, this correlation was not statistically significant with Bonferroni’s correction (Rho = 0.18, P = 0.02; Table 2). We observed no statistically significant correlation between expression of PfEMP1 containing CIDRα3.1 and CIDRα3.2 predicted to bind CD36 (primer CIDRa3.1/2) and Ang2 plasma levels or ADAM17 plasma levels (Table 2).

Next, we evaluated the Ang2 plasma level in relation to the correlated components. As the Bonferroni’s correction applied in the univariate analysis is quite conservative, we chose to include all variables correlated with a P value below 0.05 to include testing of possible false negatives, Hb, lactate, parasitaemia, Blantyre coma score, CIDRα1.4, CIDRα1.1, and ADAM17 in a general multiple linear regression analysis with backwards elimination of covariates (controlled for age and location). We found that Hb, lactate, CIDRα1.4, and ADAM17 were statistically significant explanatory variables for Ang2 plasma levels (Table 3). We evaluated the correlations found between ADAM17 plasma levels and age, HRP2, Ang2 plasma levels, CIDRα3.1/2 expression, and expression of CIDRα1.1 in a general multiple linear regression analysis with backwards elimination of covariates. We found that CIDRα1.1 expression was not a statistically significant explanatory variable for ADAM17 levels, while age and Ang2 levels were statistically significant explanatory variables (Table 4).

## Discussion

The pathology of malaria is the result of a complex interplay between inflammatory response, endothelial activation, parasite sequestration, and loss of erythrocytes. An increased amount of cytokines released by ADAM17 and endothelial activation with associated increase in plasma levels of Ang2 have been reported in malaria patients^23^. Parasites expressing EPCR-binding PfEMP1 are associated with SM^6^,^7^,^28^, and expression of group A PfEMP1s (EPCR and non-EPCR-binding PfEMP1) has been correlated with increased Ang2 plasma levels^29^.

In this study, we investigated the plasma levels of ADAM17 and Ang2 in children in north-eastern and north-western Tanzania hospitalized with malaria infections in relation to PfEMP1 expression, and the clinical parameters; Hb, lactate, Blantyre coma score, and parasitaemia. We found ADAM17 plasma levels elevated in hospitalized children with malaria compared with children with asymptomatic malaria infections. However, the ADAM17 plasma levels were similar between children with UM, SM, and other infectious diseases. During malaria convalescence, ADAM17 plasma levels markedly declined to levels similar to those of asymptomatic malaria-infected children. The increased ADAM17 concentrations in the plasma may be part of a general inflammatory response to infection and seemed to be unspecific.

In line with previous studies, Ang2 plasma levels were increased in SM patients compared with UM patients and non-malarial patients^23^,^29^. Ang2 levels in SM cases appeared higher in Magu than in Korogwe, while the UM cases had similar Ang2 levels. Ang2 levels, unlike ADAM17, correlated with markers of disease severity, such as Blantyre coma score, Hb- and lactate levels in children hospitalized with malaria. We also observed a weak correlation between Ang2 and ADAM17. Ang2 is released to the plasma from WPBs of activated endothelial cells^21^, and ADAM17 is co-localized with Ang2 in the WBPs^26^, which could explain this correlation. However, as ADAM17 plasma levels seem to be elevated as a response to disease in general, whereas Ang2 plasma levels are particularly increased in SM, it seems unlikely that the increase of ADAM17 plasma levels was primarily due to activation of endothelial cells and subsequent release of WPB. ADAM17 sheds EPCR, and loss of EPCR from the endothelial cell surface or of EPCR function may lead to a rise of plasma Ang2^30^. However, the origin of ADAM17 in plasma is unknown. Secretion of enzymatically active ADAM17 on extracellular microparticles has been found in the plasma and associated with disease activity of anti-neutrophil cytoplasmic antibody (ANCA)-associated vasculitis^26^. ANCA-associated vasculitis, as malaria, features an excessive inflammatory response, which leads to the shedding of adhesion molecules and cytokines, such as TNFα^31^,^32^.

ADAM17 sheds EPCR, and thus the increased plasma ADAM17 during acute malaria infections may have consequences for the survival of EPCR-binding parasites and for regulation of inflammation, including increased levels of plasma Ang2. However, ADAM17 plasma levels were not associated with reduced expression of EPCR-binding PfEMP1. Rather, expression of PfEMP1 with CIDRα1.1 domains was weakly correlated with ADAM17 plasma levels, although this correlation was not statistically significant when correcting for multiple testing, and lost after adjusting for age by general multiple linear regression analysis. This suggests that PfEMP1 expression and ADAM17 levels in the plasma are not directly affecting each other during malaria.

Previously, Abdi *et al*., showed that Ang2 plasma levels were associated with group A-like PfEMP1 expression and rosetting^29^, and that Ang2 levels were associated with development of the specific SM clinical manifestations RD and CM. A-type PfEMP1 expression was only associated with CM and not RD, and Abdi *et al*. concluded that Ang2 plasma levels and group A-like PfEMP1 expression were independently associated with impaired consciousness and CM^29^. Using a comprehensive set of *var* type specific primers we found that Ang2 levels are specifically correlated with expression of *var* genes encoding PfEMP1s predicted to bind EPCR. DBLα1 domain expression measured with primers targeting group A PfEMP1s (DBLα1all) correlated with Ang2, similarly to what Abdi *et al*. reported, while further division of the group A showed that only the subtype of group A (targeted by primer DBLα2/1.1/4/7) genes predicted to bind EPCR correlated with Ang2, and expression of group A PfEMP1 predicted not to bind EPCR did not correlate with Ang2. Furthermore, measurements of additional *var* genes encoding PfEMP1s not predicted to bind EPCR, including group B PfEMP1 (UPSB), PfEMP1 containing CIDRα3.1/2 predicted to bind CD36 or chondroitin sulfate A (VAR2CSA), did not correlate with plasma levels of Ang2.

We did not find a correlation between the transcript levels of all analysed CIDRα1 domains predicted to bind EPCR, so caution is warranted in interpreting the found correlations, but it could be due to less prevalence of CIDRα1 expression of these subtypes in the infecting parasites. Our measurements of PfEMP1 expression are not able to fully account for the increase in Ang2 observed in SM, and cannot alone explain the increase in Ang2 observed in SM, as underlined by our multiple linear regression analysis of Ang2 levels. So while Abdi *et al*. reports that endothelial activation and group A expression are independently associated with CM, we find that part of the Ang2 plasma level which is increased in SM, is associated with EPCR-binding PfEMP1 expression, even when including other factors associated with increased Ang2 in the analysis, such as lactate and Hb.

Recently, an analysis of full-length *var* transcripts in UM and SM groups revealed that they mainly differ in relation to an increased expression of EPCR-binding PfEMP1 in SM, but with no major differences in *var* transcripts between SMA and CM groups^33^.

In summary, these data suggest that ADAM17 plasma levels increase in response to malaria infection similarly to that of other infectious diseases and that the association between Ang2 levels and *var* gene transcript levels, although weak, is specific for EPCR-binding PfEMP1s. Furthermore, PfEMP1s predicted to bind EPCR may be associated with the endothelial activation accompanying SM, reinforcing the notion that EPCR-binding PfEMP1s are linked to SM pathogenesis (Supplementary Fig. S3).

## Methods

### Sample collection

A total of 213 children admitted to Korogwe and Magu District Hospitals, in north-eastern and north-western Tanzania, respectively, were enrolled as part of an ongoing monitoring of malaria at the hospitals. 181 children were blood smear positive for *P. falciparum*. For 19 children admitted to the Korogwe District Hospital with malaria, blood samples were drawn at admission and at follow-up between 2 and 12 weeks after admission. None were malaria positive at follow-up by rapid diagnostic tests. 32 of the children admitted to Korogwe District Hospital with infectious diseases other than malaria were included as non-malaria hospitalized children and also a part of a previous study^34^ (Supplementary Table S1). The patient samples included in this study were selected from a database to reflect the main clinical manifestations associated with high mortality; (I) CM was diagnosed if the Blantyre coma score was <3^35^), (II) SMA if the haemoglobin (Hb) level was <5 g/dl, (III) if there were clinical signs of RD (Kussmaul deep breathing and/or chest indrawing), or (IV) if hyperparasitaemia was observed (>200,000 parasites/μl). Children fulfilling any of the above criteria were categorized as SM. Children not fulfilling any of these criteria were categorized as UM. Plasma samples from six non-hospitalized children as part of a malariometric survey in the Korogwe district with asymptomatic malaria were included in the study.

### Ethics statement

Written informed consent was obtained from the parents or guardian of all the study participants. All patients received treatment according to the national guidelines. The studies and experiments were reviewed and approved by the National Institute for Medical Research, Tanzania (reference no. NIMR/HQ/R.8c/Vol.II/436), and the methods in this study were perfomed in accordance with the approved guidelines.

### Detection of plasma proteins

The plasma samples were diluted in phosphate buffered saline (PBS) +1% bovine serum albumin (BSA), between 1:2 and 1:6 for ADAM17 and 1:5 for Ang2, and ADAM17 and Ang2 plasma levels were detected using DuoSet ELISA Development kit (R&D Systems) according to manufacturer’s instructions. A four parameter logistic curve was fitted to a serial dilution of a recombinant protein standard of known concentration for each ELISA plate, in order to convert optical densities to protein concentration.

The PfHRP2 plasma levels were determined using an ELISA assay as previously described^36^, with the following modifications: Maxisorp plates (NUNC, Roskilde, Denmark) were coated with 1.0 μg/mL immunoglobulin M monoclonal anti-HRP2 antibody (MPFM-55a, Immunology Consultants Laboratories, Portland, USA) diluted in PBS and incubated overnight at 4 °C. Plates were blocked with 3% skimmed milk in PBS for 2 hours. Between each step the plates were washed in 0.05% Tween-20 and PBS. Plasma samples were diluted 1:64 in 1% skimmed milk and PBS, added to the plates and incubated for 2 hours. Secondary antibody conjugated with horseradish peroxidase (MPFG-55P, Immunology Consultants Laboratories; 0.2 μg/mL diluted in 1% skimmed milk and PBS) was added to each well and incubated for 1 hour at room temperature. Substrate, TMB ease (Kem-En-Tec Diagnostics), was added and the reaction was stopped with 0.2 M H_2_SO_4_. The plates were read using a plate reader at an OD of 490. A serial dilution of a recombinant HRP2 protein (Cat no MBS5303860, MyBioSource, San Diego, USA) was made for each plate in order to convert optical densities to protein concentration as for ADAM17 and Ang2.

### *Var* transcript abundances

Data of transcript abundances of *var* gene subtypes were generated using quantitative PCR by Mkumbaye *et al*., (*submitted*) as previously described^7^, although using redesigned primers targeting loci specific for different PfEMP1 domains. The qPCR primer sets were optimized for optimal coverage of *var* genes encoding specific subclasses of DBL and CIDR domains. Supplementary Table S2 provides an overview of the estimated *var* type coverage of the primers used in this study. qPCR was performed in 20-μL reactions using QuantiTect SYBR Green PCR master mix (Qiagen) with the Rotorgene thermal cycler system (Corbett Research), and the transcript abundance was determined relative to the averaged transcript abundance of *seryl-tRNA synthetase* and *fructose-bisphosphate aldolase*, as previously described^7^. Summation of *var* transcript levels reported by primers targeting *var* transcripts encoding PfEMP1 with similar receptor binding specificity was done for: PfEMP1 with CIDRα1 domains predicted to bind EPCR (CIDRa1.all): primer sets CIDRa1.1, CIDRa1.4, CIDRa1.5, CIDRa1.6, CIDRa1.7, and CIDRa1.8; Group A CIDRα1 domains predicted to bind EPCR (CIDRa1.A): primer sets CIDRa1.4, CIDRa1.5, CIDRa1.6, and CIDRa1.7; and DC8 (CIDRα1.DC8): primer sets CIDRa1.1 and CIDRa1.8.

### Statistical analysis

Normally distributed variables, such as age, were compared by Welch’s Two Sample t-test. For comparing acute and convalescent ADAM17 plasma levels, Wilcoxon’s paired signed rank test was used to calculate statistical significance. For comparisons of multiple groups, the non-parametric Kruskal-Wallis test was used, while differences between groups were calculated by pairwise comparisons using Dunn’s test with Bonferroni’s correction. For univariate correlations, Spearman’s rank correlation was used to determine the correlation coefficient Rho. Bonferroni’s correction was applied to the spearman’s rank correlations; ADAM17 and Ang2 were considered independent tests, so 24 components were included in the correction for each (DBLα1all, CIDRα1.all, CIDRα1.A, and CIDRα1.DC8 were not counted as they represent summations of primers that are included by themselves) and thus correlations were considered statistically significant when the P-value was below 0.002. General linear regression analysis was performed, in which we included the univariate correlations with a P value below 0.05. Backwards elimination was performed with multiple iterations, each time removing the variable with the highest P-value above the significance level, until only statistically significant explanatory variables remained. Statistical analyses were performed using the statistical software R. A P-value below 0.05 was considered statistically significant.

## Additional Information

**How to cite this article**: Petersen, J. E. V. *et al*. Plasma Ang2 and ADAM17 levels are elevated during clinical malaria; Ang2 level correlates with severity and expression of EPCR-binding PfEMP1. *Sci. Rep.* **6**, 35950; doi: 10.1038/srep35950 (2016).

**Publisher’s note:** Springer Nature remains neutral with regard to jurisdictional claims in published maps and institutional affiliations.

## Supplementary Material

Supplementary Information

## Figures and Tables

**Table 1:** Clinical characteristics of patients.

*Patient characteristics*	*Village infections* (*VI*) (*n* = *6*)	*Uncomplicated malaria* (*UM*) (*n* = *39*)	*Severe malaria* (*SM*) (*n* = *123*)	*Non-malaria hospitalized* (*n* = *32*)	*Acute SM/Convalescent (*n* = *19*)
*Age*(*years*)*, mean* [*IQR*] *Min-max*	9.4 [5.8–11.5] 5.2–16.9	3.0 [2.0–4.0] 0.2–7.0	2.8 [1.6–4.0] 0.1–9.2	1.5 [0.6–2.2] 0.2–4.9	2.5 [1.3–3.5] 0.3–4.2
*Sex, n* (*M*/*F*)	3/3	16/23	60/63	21/11	10/9
*Location, n* (*Korogwe*/*Magu*)	NA	32/7	75/48	32/0	19/0
*Parasitaemia, median* (*p*/*μL*) [*IQR*]	989 [363–1,650]	12,500 [5,080–45,000]	272,000 [35,000–512,200]	NA	17,000 [9,900–427,000]
*HRP2, median* (*μg*/*mL*) [*IQR*]	435 [158–3371]	1662 [92–3215]	3989 [868–8013]	0 [0–0]	1715 [1020–2380]
*Temperature, median* (*°C*) [*IQR*]	37.1 [36.8–38.2]	38.0 [37.6–39.7]	38.4 [37.7–39.4]	39.3 [38.9–39.8]	38.8 [38.1–39.1]
*Haemoglobin, mean* ± *sd* (*g*/*dL*) *Min-max*	11.3 ± 2.6 6.7–14.7	9.9 ± 2.0 8.1–20.2	6.16 ± 2.3 2.4–11.3	9.1 ± 1.6 5.6–12.2	5.9 ± 2.9 6.7–14.7
*Blantyre coma score, mean* [*IQR*]*Min-max*	NA	5 [5–5] 5–5	3.6 [2–5] 0–5	4.8 [5–5] 1–5	4.5 [5–5] 2–5
*Lactate, mean* ± *sd* (*mM*) *Min-max*	NA	3.4 ± 1.8 1.4–9.0	5 ± 3.4 0.9–17.2	3.1 ± 1.2 1.5–6.1	5.5 ± 3.1 1.1–13.1
*Mortality, n* (*%*)	0	0	9 (7)	1 (3)	0
*Convalescent sample, mean* (*weeks*) *Min-max*	NA	NA	NA	NA	6.3 2.4–10.7

^*^For 19 SM cases a convalescent plasma sample was taken.

IQR = Inter Quartile Range, sd = Standard deviation.

**Table 2:** Correlations of ADAM17 and Ang2 Plasma levels.

	*ADAM17*		*Ang2*	
	*Rho*	*P value*	*Rho*	*P value*
*Age*	−0.24	0.0013*	−0.014	0.86
*Hb*	−0.10	0.15	−0.39	0.0000006*
*Lactate*	0.08	0.34	0.35	0.0001*
*Parasitaemia*	−0.05	0.50	0.15	0.06
*HRP2*	0.17	0.03	0.07	0.42
*Blantyre score*	−0.02	0.84	−0.29	0.0002*
*Ang2*	0.18	0.02		
*DBLa1all*	0.1	0.21	0.24	0.002*
* DBLa2/1.1/2/4/7*	0.10	0.19	0.25	0.002*
* DBLa1.5/6/8*	0.09	0.23	0.09	0.27
*CIDRa1.all*	0.08	0.34	0.17	0.04
* CIDRa1.A*	−0.03	0.75	0.18	0.02
* CIDRa1.4*	0.02	0.73	0.18	0.02
* CIDRa1.5*	−0.08	0.31	0.05	0.50
* CIDRa1.6*	−0.03	0.71	0.07	0.37
* CIDRa1.7*	−0.09	0.23	0.08	0.28
*CIDRa1.DC8*	0.08	0.28	0.15	0.06
* CIDRa1.1*	0.18	0.02	0.25	0.001*
* CIDRa1.8*	−0.04	0.56	0.07	0.40
*CIDRa3.1/2*	0.15	0.05	0.10	0.23
*UPSB*	0.03	0.69	0.03	0.72
*VAR2CSA*	−0.11	0.16	−0.02	0.78
*Var3*	0.13	0.12	0.02	0.83
*CIDRd*	0.09	0.21	−0.02	0.81
*CIDRg*	0.04	0.59	−0.04	0.59
*DBLz*	0.03	0.71	0.10	0.19
*DBLe*	0.03	0.66	−0.03	0.75
*DC5*	0.03	0.74	−0.05	0.51

^*^Statistically significant correlations when applying Bonferroni’s correction for multiple testing with 24 independent tests for ADAM17 and Ang2 each (P ≤ 0.002 considered statistically significant, DBLα1all, CIDRα1.all, CIDRα1.A, and CIDRα1.DC8 were not counted as they represent summations). CIDRα1.all = Sum of all primers targeting transcripts encoding CIDRα1s predicted to bind EPCR. CIDRa1.A EPCR = Sum of all transcript abundances reported by primers targeting genes encoding Group A CIDRα1s predicted to bind EPCR. CIDRα1.DC8 = Sum of all reported transcripts encoding Group B/A CIDRα1s predicted to bind EPCR.

Spearman’s rank correlation coefficients (Rho) calculated for uncomplicated and severe acute malaria patients.

**Table 3:** Statistically significant explanatory variables in a multiple linear regression analysis of transformed Ang2 levels in hospitalized malaria patients.

*Model*	*Estimate*	*P*
*Hb* (*g/dL*)	−0.03	0.001
*Lactate*	0.02	0.004
*CIDRα1.4*	0.003	0.004
*Log10* (*ADAM17*) (*pg*/*ml*)	0.15	0.02
*Location 2*	0.2	0.0001

**Table 4:** Statistically significant explanatory variables in a multiple linear regression analysis of transformed ADAM17 levels in hospitalized malaria patients.

*Model*	*Estimate*	*P*
*Age* (*years*)	−0.05	0.002
*Log10* (*Ang2* *pg*/*ml*)	0.25	0.005

## References

[b1] World Health Organization. *World Malaria Report 2015*. *World Health*, doi: ISBN 978 92 4 1564403 (2015).

[b2] Miller L. H., Baruch D. I., Marsh K. & Doumbo O. K. The pathogenic basis of malaria. Nature 415, 673–679 (2002). 11832955 10.1038/415673a

[b3] Turner L. . Severe malaria is associated with parasite binding to endothelial protein C receptor. Nature 498, 502–505 (2013). 23739325 10.1038/nature12216 PMC3870021

[b4] Lavstsen T., Salanti A., Jensen A. T. R., Arnot D. E. & Theander T. G. Sub-grouping of Plasmodium falciparum 3D7 var genes based on sequence analysis of coding and non-coding regions. Malar. J. 2, 27 (2003). 14565852 10.1186/1475-2875-2-27 PMC222925

[b5] Rask T. S., Hansen D. A., Theander T. G., Gorm Pedersen A. & Lavstsen T. Plasmodium falciparum erythrocyte membrane protein 1 diversity in seven genomes–divide and conquer. PLoS Comput. Biol. 6, e1000933 (2010). 20862303 10.1371/journal.pcbi.1000933 PMC2940729

[b6] Lau C. K. Y. . Structural conservation despite huge sequence diversity allows EPCR binding by the pfemp1 family implicated in severe childhood malaria. Cell Host Microbe 17, 118–129 (2015). 25482433 10.1016/j.chom.2014.11.007 PMC4297295

[b7] Lavstsen T. . Plasmodium falciparum erythrocyte membrane protein 1 domain cassettes 8 and 13 are associated with severe malaria in children. Proc. Natl. Acad. Sci. 109, E1791–E1800 (2012). 22619319 10.1073/pnas.1120455109 PMC3387094

[b8] Avril M. . A restricted subset of var genes mediates adherence of Plasmodium falciparum-infected erythrocytes to brain endothelial cells. Proc. Natl. Acad. Sci. 109, E1782–E1790 (2012). 22619321 10.1073/pnas.1120534109 PMC3387091

[b9] Claessens A. & Rowe J. A. Selection of Plasmodium falciparum parasites for cytoadhesion to human brain endothelial cells. J. Vis. Exp. e3122, 10.3791/3122 (2012). 22230803 PMC3369769

[b10] Wajant H., Pfizenmaier K. & Scheurich P. Tumor necrosis factor signaling. Cell Death Differ. 10, 45–65 (2003). 12655295 10.1038/sj.cdd.4401189

[b11] Sundgren-Andersson A. K., Ostlund P. & Bartfai T. IL-6 is essential in TNF-alpha-induced fever. Am. J. Physiol. 275, R2028–R2034 (1998). 9843893 10.1152/ajpregu.1998.275.6.R2028

[b12] Kwiatkowski D. . TNF concentration in fatal cerebral, non-fatal cerebral, and uncomplicated Plasmodium falciparum malaria. Lancet 336, 1201–1204 (1990). 1978068 10.1016/0140-6736(90)92827-5

[b13] Moss M. L. . Structural features and biochemical properties of TNF-alpha converting enzyme (TACE). J. Neuroimmunol. 72, 127–129 (1997). 9042103 10.1016/s0165-5728(96)00180-4

[b14] Scheller J., Chalaris A., Garbers C. & Rose-John S. ADAM17: A molecular switch to control inflammation and tissue regeneration. Trends in Immunology 32, 380–387 (2011). 21752713 10.1016/j.it.2011.05.005

[b15] Tsakadze N. L. . Tumor necrosis factor-alpha-converting enzyme (TACE/ADAM-17) mediates the ectodomain cleavage of intercellular adhesion molecule-1 (ICAM-1). J. Biol. Chem. 281, 3157–3164 (2006). 16332693 10.1074/jbc.M510797200

[b16] Qu D., Wang Y., Esmon N. L. & Esmon C. T. Regulated endothelial protein C receptor shedding is mediated by tumor necrosis factor-? converting enzyme/ADAM17. J. Thromb. Haemost. 5, 395–402 (2007). 17155946 10.1111/j.1538-7836.2007.02347.x

[b17] Moxon C. A. . Loss of endothelial protein C receptors links coagulation and inflammation to parasite sequestration in cerebral malaria in African children. Blood 122, 842–851 (2013). 23741007 10.1182/blood-2013-03-490219 PMC3731936

[b18] Sampath S. . Plasmodium falciparum adhesion domains linked to severe malaria differ in blockade of endothelial protein C receptor. Cell. Microbiol. 17, 1868–1882 (2015). 26118955 10.1111/cmi.12478 PMC4661071

[b19] Petersen J. E. V. . Protein C system defects inflicted by the malaria parasite protein PfEMP1 can be overcome by a soluble EPCR variant. Thromb. Haemost. 114, 1038–1048 (2015). 26155776 10.1160/TH15-01-0018 PMC8142631

[b20] Gillrie M. R. . Diverse functional outcomes of Plasmodium falciparum ligation of EPCR: Potential implications for malarial pathogenesis. Cell. Microbiol. 17, 1883–1899 (2015). 26119044 10.1111/cmi.12479 PMC4661070

[b21] Fiedler U. . The Tie-2 ligand Angiopoietin-2 is stored in and rapidly released upon stimulation from endothelial cell Weibel-Palade bodies. Blood 103, 4150–4156 (2004). 14976056 10.1182/blood-2003-10-3685

[b22] Lovegrove F. E. . Serum angiopoietin-1 and -2 levels discriminate cerebral malaria from uncomplicated malaria and predict clinical outcome in African children. PLoS One 4 (2009). 10.1371/journal.pone.0004912 PMC2657207 19300530

[b23] Yeo T. W. . Angiopoietin-2 is associated with decreased endothelial nitric oxide and poor clinical outcome in severe falciparum malaria. Proc. Natl. Acad. Sci. USA 105, 17097–17102 (2008). 18957536 10.1073/pnas.0805782105 PMC2575222

[b24] Hakanpaa L. . Endothelial destabilization by angiopoietin-2 via integrin β1 activation. Nat. Commun. 6, 5962 (2015). 25635707 10.1038/ncomms6962 PMC4316742

[b25] Dreymueller D. . Lung endothelial ADAM17 regulates the acute inflammatory response to lipopolysaccharide. EMBO Mol. Med. 4, 412–423 (2012). 22367719 10.1002/emmm.201200217 PMC3403298

[b26] Bertram A. . Circulating ADAM17 Level Reflects Disease Activity in Proteinase-3 ANCA-Associated Vasculitis. J. Am. Soc. Nephrol, 10.1681/ASN.2014050477 (2015). PMC4625662 25788529

[b27] Miller L. H., Ackerman H. C., Su X. & Wellems T. E. Malaria biology and disease pathogenesis: insights for new treatments. Nat. Med. 19, 156–167 (2013). 23389616 10.1038/nm.3073 PMC4783790

[b28] Turner L. . Severe malaria is associated with parasite binding to endothelial protein C receptor. Nature 498, 502–505 (2013). 23739325 10.1038/nature12216 PMC3870021

[b29] Abdi A. I. . Plasmodium falciparum antigenic variation: relationships between widespread endothelial activation, parasite PfEMP1 expression and severe malaria. BMC Infect. Dis. 14, 170 (2014). 24674301 10.1186/1471-2334-14-170 PMC3986854

[b30] Bae J. S. & Rezaie A. R. Thrombin upregulates the angiopoietin-Tie2 Axis: Endothelial protein C receptor occupancy prevents the thrombin mobilization of angiopoietin 2 and P-selectin from Weibel-Palade bodies. J. Thromb. Haemost. 8, 1107–1115 (2010). 20180904 10.1111/j.1538-7836.2010.03812.x PMC2891946

[b31] Ohlsson S., Wieslander J. & Segelmark M. Circulating cytokine profile in anti-neutrophilic cytoplasmatic autoantibody-associated vasculitis: prediction of outcome? Mediators Inflamm. 13, 275–283 (2004). 15545059 10.1080/09629350400003100 PMC1781567

[b32] Noronha I. L., Krüger C., Andrassy K., Ritz E. & Waldherr R. *In situ* production of TNF-alpha, IL-1 beta and IL-2R in ANCA-positive glomerulonephritis. Kidney Int. 43, 682–692 (1993). 8455368 10.1038/ki.1993.98

[b33] Jespersen J. S. . *Plasmodium falciparum var* genes expressed in children with severe malaria encode CIDRα1 domains. EMBO Mol. Med. 8, 839–850 (2016). 27354391 10.15252/emmm.201606188 PMC4967939

[b34] Hansson H. H. . Haplotypes of the endothelial protein C receptor (EPCR) gene are not associated with severe malaria in Tanzania. Malar. J. 14, 474 (2015). 26620701 10.1186/s12936-015-1007-6 PMC4666078

[b35] Molyneux M. E., Taylor T. E., Wirima J. J. & Borgstein A. Clinical features and prognostic indicators in paediatric cerebral malaria: a study of 131 comatose Malawian children. Q. J. Med. 71, 441–459 (1989). 2690177

[b36] Kariuki S. M. . Value of plasmodium falciparum histidine-rich protein 2 level and malaria retinopathy in distinguishing cerebral malaria from other acute encephalopathies in Kenyan children. J. Infect. Dis. 209, 600–609 (2014). 24041795 10.1093/infdis/jit500 PMC3903374

