# Supplementary material for: Plasma Ang2 and ADAM17 levels are elevated during clinical malaria; Ang2 level correlates with severity and expression of EPCR-binding PfEMP1
Source: Sci Rep. 2016 Oct 27;6:35950. doi: 10.1038/srep35950 (PMC5082358; doi:10.1038/srep35950)
Supplement: Supplementary Information [file srep35950-s1.pdf]

Supplementary information

Plasma Ang2 and ADAM17 levels are elevated during clinical malaria; Ang2 level correlates with severity and expression of EPCR-binding PfEMP1

Jens E.V. Petersen*[1], Sixbert I. Mkumbaye*[2], Anna V. Vaaben[1], Alphaxard Manjurano[3], Eric Lyimo[3], Reginald A. Kavishe[2], Steven B. Mwakalinga[2], Jacklin Mosha[3], Daniel T.R. Minja[4], John P. Lusingu[4], Thor G. Theander[1], Thomas Lavstsen[1], Christian W. Wang[1].

	Septicemia or meningitis (n=9)	***Pneumonia or submandibular swelling (n=14)****	***Gastroenteritis or urinary tract infections (n=9)****
Age, mean [IQR] (Years)<br>*Min-max*	1.8 [1.0 - 2.6]<br>0.5 – 3.8	1.4 [0.5 - 1.8]<br>0.16 - 4.9	1.2 [0.3- 2.1]<br>0.16 - 2.9
Sex M/F	7/2	7/7	7/2
Temperature, median (°C)<br>*[IQR]*	38.9<br>[38.9 - 39.5]	39.4<br>[38.9 - 39.8]	39.2<br>[38.9 - 39.9]
Haemoglobin, mean±sd (g/dL)<br>*Min-max*	8.6 ± 1.8<br>5.6 - 11.1	8.7 ± 1.5<br>6.5 - 12.1	9.9 ± 1.6<br>7.7 - 12.2
Lactate, mean ± sd (mM)<br>*Min-max*	3.9 ± 1.3<br>2.4 - 6.1	2.9 ± 1.2<br>1.5 - 6.0	2.8 ± 1.0<br>1.5 - 4.5
Died	1/9	0/14	0/9

*Patients suffering from both pneumonia and gastroenteritis were grouped in the “pneumonia or submandibular swelling” group

Table S1. Non-malaria patient characteristics

**Target**<br>***Primer name*** **(Coverage^)**	**Forward primer (cocktail)**	**Reverse primer (cocktail)**
DBLα1, group A<br><br>*DBLa1all* (92%)	TTGGGAAATGTRTTRGTTACAGCAAA<br>TTGGGAAATGTGTTAGTTATGGCAAA<br>TTGGGGAATTTGTTAGTTATGGCAAG<br>TTGGGGAACCTATTAGTTATGGCAA<br>TTAGGAAATATATTGGTAGCAGCAA<br>TTAGGAAATATCTTGGTCACAGCAA	CCTATATCNGCAAAACTKCKWGC
DBLα of EPCR-binding DC8 and group A<br><br>*DBLa2/1.1/1.2/1.4/1.7* (82%)	GATTAYGTBCCTCAATTTT AMGWTGGT	TACAATCATATCCATTAWGACTACAA<br>TCACAATCGCATCCATTATGACTACAA
DBLα non-EPCR binding group A<br><br>*DBLa1.5/1.6/1.8* (74%)	GATTAYGTBCCTCAATTTTAMGWTGGT	TTTTAGTACAATCATAACCATCACCA<br>GATTTGTTTTWTTACAATCGTAACCCTC<br>ACAATCCTCACCATCACCACTACAAT<br>CGTGATATATCTGTTTKAGTACAATC<br>GATCTGTTCGTTTACAATCGTAACCCTC
CIDRα1.4<br><br>*CIDRa1.4* (78%)	AACTATCAAAAATGGGAATGCTATTA<br>AACTATGAACAATGGAAATGCTATTA<br>AACTATCAAAAATGGAATTGCTATTA<br>AACTATGAAAACTGGCAATGCTATTA<br>AACAATCAAATATGGAAATGCTATTA	TTTCCCACTTTATAGTGTCTATTA<br>TTTCCCATTTTATAGTGTCTATTA<br>TTTCCCACTTTATACTGTCTATTA<br>TTTCCCAGTTTATAGTGTCTATTA<br>TTTCCCACTCTATAGAGTCTATTA
CIDRα1.5*<br><br>*CIDRa1.5a* (93%)<br><br>*CIDRa1.5b* (96%)	GATTTATGGATTAAGAATTTATTAAG<br>GATTTGTGGGTTACGAATTTATTAAG<br>GATTTGTGGGTTACATATTTATTAAG<br><br>ACGATACTATAGACTGGAAATACG<br>ATTGGGAAWATAAACTTAAGACCTG<br>TGGATACTACAGATTGGGATCGTA	TAATTCATCCGTAAATTTCTTCCA<br>CAAATCTTCCTTAAGTTTTTTCCA<br>TAATTCATCCGTAAATTGATTCCA<br>CAAATCTTCTTTAAGTTTTTTCCA<br><br>AACCCATTGTTCAAAACATTTACA<br>AACCCATTTATCAAAACACGTACA<br>AACCCATTTATCAAAACACATACA
CIDRα1.6<br><br>*CIDRa1.6b* (100%)	ATAATACTAATGTSACGGATTGT	CAGTTTCTTTATACTATCCCATTC<br>ACATCCTTTATACTACCCCATTCC<br>AATTCCTTTATACTCTTCCATTCTG
CIDRα1.7<br><br>*CIDRa1.7* (99%)	CGGAAACTATAACGTGGAACGATAA<br>CGGAAACTATAAGGTGGAACGATAA<br>CGGAAACTATAACGTGGAAAGATAA<br>GGATACTATAATGTGGAATGATAAA	TAGTTTCTTTATACTATTCCATTC<br>TAGTTCCTTTATACTATTCCATTC<br>TAGTTTCTTTATATTATTCCATTC<br>TAGTTTCTTTATACTACTCCATTC<br>TAATTCCTTTATATTATTCCATTC
CIDRα1.1<br><br>*CIDRa1.1* (95%)	TGGGAACATCAACTTAAGGATTGCATA<br>TGGGAACATCAACTTAAGAATTGCATA<br>TGGGAACATGAACTTAAGGATTGCATA	TAAATCTTYCNTAAATTGATHCCAT
CIDRα1.8*<br><br>*CIDRa1.8a* (100%)<br><br>*CIDRa1.8b* (100%)	ATAATTGTGAAATGAAAGGTTCA<br><br>AATAGACAGTATAATGTGGGAA<br>AAAGGATACTATAAAGTGGGAA	TATGCAMTTCTTAAGTTTGGTTTCC<br><br>CAAAACATWTACAATTTTCGTTACA

CIDRα3.1/2<br><br>*CIDRa3.1/2* (75%, 17% of CD36-binding CIDRa2-6)	AHWWVCAAAAGACRTWCHATRATTT<br>AHWWVCAAAAGACRTTCAATCCT<br>ARAAAGTAAAGGATTATGTWGRTTT	TTTTTGTTCTCCAATRTATRGAATC
UPSB<br>*UPSB* (Not applicable)	1. Rottmann et al., 2006	1. Rottmann et al., 2006
VAR2CSA/DBL3x<br>*T12/T13* (100%)	2. Sander et al., 2009	2. Sander et al., 2009
Var3/DBLe8<br>*DBLe8* (100%)	3. Lavstsen et al., 2012	3. Lavstsen et al., 2012
CIDRδ<br><br>*CIDRd* (75%)	TAAATGTAACTTAGATGTATGTGAAC<br>TAAATGTAACTTACATGTATGTGAAC<br>TAAATGTAACTTAGACGTATGTGAAC<br>TAAATGTTACTTAGATGTATGTGAAC<br>TAAATGTAACTTAGATATATGTGAAC<br>TAAATGTAAGTTAGATGTATGTGAAC	AATACTTTAACCAACGTTTAATCAATAC<br>AATACTTTAACCAACGCTTAATCAATAC<br>AATACTGCAACCAACGTTTAATCAATAC<br>AATGCTCTAACCAACGTTTAATGAATAC<br>AATACATCAACCAACGCTTAATCAATAC<br>AATATTCTAACCACCGTTTAAATAGTAC
CIDRγ3.1<br><br>*CIDRg3.1* (100%)	TATGTATATGCTGATGAACGT	TTCTATCCATTTTTCTAAACATTC
DBLζ_all**<br><br>*DBLz2a* (19%)<br><br>*DBLz2b* (34%)<br><br>*DBLz2c* (31%)<br><br>*DBLz3* (34%)<br><br>*DBLz4* (93%)<br><br>*DBLz5* (94%)<br><br>*DBLz6* (81%)	CCTCAACGTTTRAGATGGATGAAG<br><br>AGTGACCCTCCTGTGGATGATTA<br><br>ATGTRCCTCAAATACTTAGATGGATWA<br><br>AACCTCCTTATGTTGATTACATTCCACA<br>ATCCTCCTTATGATGATTATATWCCWCA<br><br>AACCTCCTGATTATGATTATATACCT<br><br>GATTATGATTATATYCCTCAACCTT<br><br>CCTGATTATGATTATATWCCYCAACCTT	ATTTTGCAAACATAYTCTCCCCATT<br><br>TGCAATAATTTTCACTCCATTCCTT<br><br>TTCTTGCAATTCACAAAAATGTTC<br><br>TTCTGACCATTCAGTCATCCATCT<br><br>CAATAATATTCACTCCATTCTTGC<br><br>TTCRCYCCATTCACTTAKCCAKCG<br><br>TTCACTCCATTCTTGCATSMAACG
DBLε_all***<br><br>*DBLe2* (80%)<br><br>*DBLe6* (76%)<br><br>*DBLe11* (91%)<br><br>*DBLe13* (85%)<br><br>*DBLe14* (85%)	AAAWTTAATWGGTTTGGRAGCAC<br>AATTTRATWGGTTTAAATGCAYACA<br><br>GTTGCATAYAATGAAGGTTATTTCCT<br>AAAACTRRACTTATGAATTSTGCCTACA<br>ATTTCTTCAATATGYCTAYACTSAAGGA<br><br>AAAGHATTACAAAAAGAYGCATAT<br>CATTACAAATATGTGCATATAATSAAG<br>AAATACAGGAATCAGCATACAACGAAG<br><br>CTGTTGCTGCAAATSAAGGATATAAT<br>CTGCTGYTGCAAATGATGCATATAAT<br><br>TTTAAYCAAGGAATWCTTTTAGGAA	GACATAATTGTTGYACTCTAGGAGRMA<br><br>ATCHGTWCCTTTAACTATATCTCCATA<br>ATCTYTACCTTTTACTATATCAGCAAT<br><br>TTCCTTTAATTAAATYASCATAATCWGCA<br>TCAGCATAACTYCTTTTCATATTTTCA<br><br>ATAATCRTAAAAACTRTATTTCAATGCAT<br><br>ATATCTSCATARTCAGCAAAACTAT
DBLβ in DC5<br><br>*DC5* (78%)	GTTGCTCCYMCTTTTTGTAATGT<br>CCCCCHCCYTTTTGTAAYGTNCC<br>TTGCACCCATTTTTTGTAATATGCC	ACCACRTTGGTCGCATCTTTGTC<br>CACTCACTATGTTGGTGYCATTTTT<br>GCCASCACTTCWACTCCNACCAC<br>YACAYTWACCACATTNGTSGM

Table-S2. **List of primers used for quantitative PCR.**

^) Predicted Coverage of genes encoding domain class (Mkumbaye et al., submitted)

*A summated transcript level of the CIDRa1.5a and b and CIDRa1.8a and b primers were used, respectively.

** The summated transcript level of transcripts encoding DBLζ domains, calculated as sum abundances reported by DBLz2+z3+z4/6 primers. Due to the considerable overlap between DBLz4 and DBLz6 primer sets, the transcript level for these domains were analyzed by using only the data from the primer reporting highest transcript level. ***A summated transcript level for all targeted genes encoding DBLε.

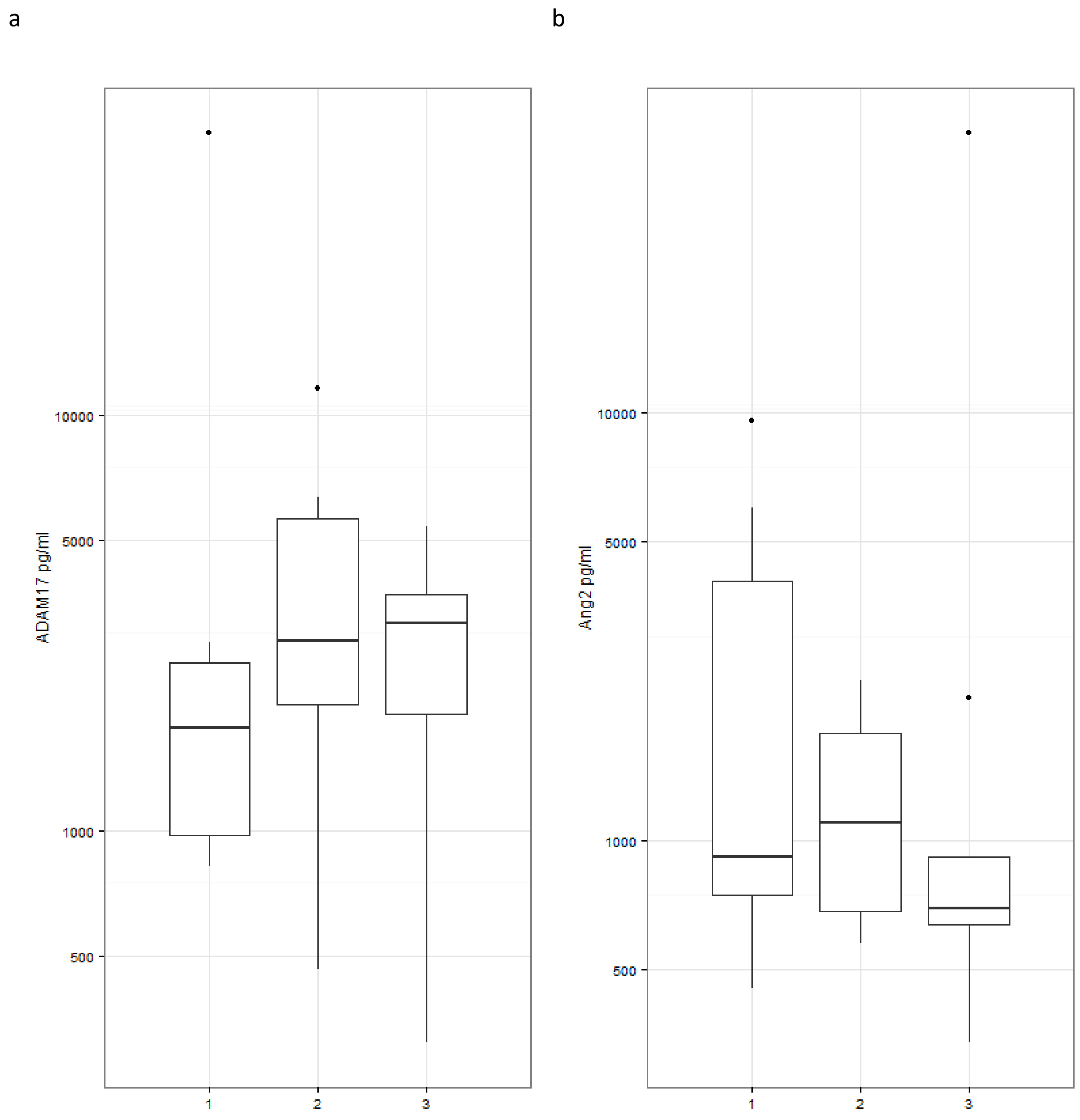

Figure S1. Boxplot of non-malaria patient groups' ADAM17 and Ang2 plasma levels. 1 is septicemia and/or meningitis (n=9), 2 is pneumonia or submandibular swelling (n=14), and 3 is gastroenteritis or urinary tract infections (n=9). **(a)** ADAM17 plasma levels; no statistically significant difference between groups (P=0.3). **(b)** Ang2 plasma levels, no statistically significant difference between groups (P=0.5). The boxplots display median, 1st quartile (Q1), and 3rd quartile (Q3). The lower whisker indicates the lowest data point above Q1 minus 1.5 times IQR, the upper whisker indicates the highest data point below 1.5 IQR plus Q3. Values below Q1 minus 1.5 IQR and above Q3 plus 1.5 IQR are indicated as points. Difference between groups was tested with Kruskal-Wallis rank sum test.

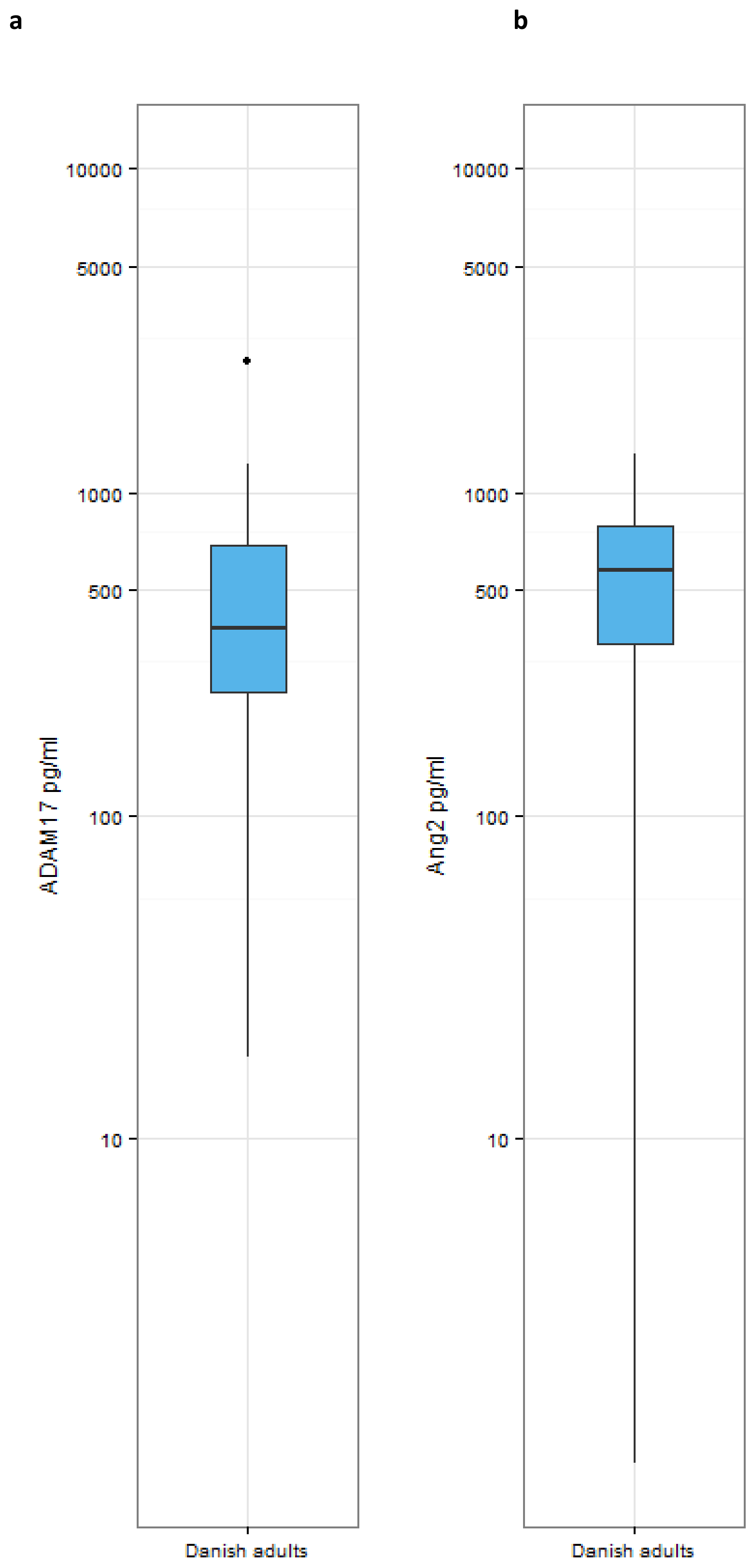

Figure S2. ADAM17 and Ang2 plasma levels in healthy Danish adults. (a) ADAM17 plasma levels of healthy Danish adults (n=39). **(b)** Ang2 plasma levels of healthy Danish adults (n=29). The boxplots display median,

1st quartile (Q1), and 3rd quartile (Q3). The lower whisker indicates the lowest data point above Q1 minus 1.5 times IQR, the upper whisker indicates the highest data point below 1.5 IQR plus Q3. Values below Q1 minus 1.5 IQR and above Q3 plus 1.5 IQR are indicated as points.

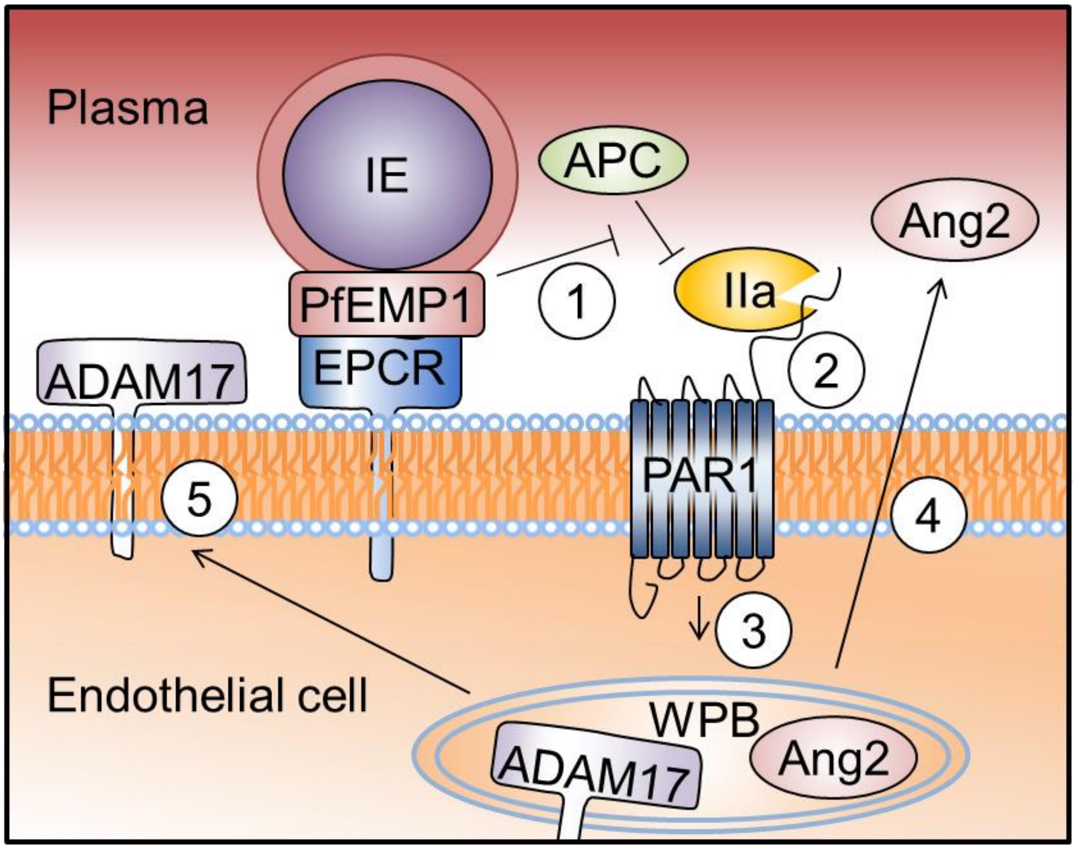

Figure S3 Proposed model of PfEMP1s effect on Ang2 release from endothelial cells. (1) PfEMP1 expressed at the surface of infected erythrocytes (IE) binds to EPCR and inhibits activated protein C (APC) binding to EPCR and thus also blocks APCs ability to inhibit Thrombin (IIa) cleavage of Protease Activated Receptor 1 (PAR1). (2) Thrombin cleavage of PAR1 activates the endothelial cell. (3) PAR1 signaling causes release of the Weibel Palade Bodies (WPBs), which contains Ang2 and ADAM17. (4) Ang2 is released to the plasma from the WPBs and (5) ADAM17 is increased on the cell surface.

References

[1] Rottmann M. *et al*. Differential expression of *var* gene groups is associated with morbidity caused by Plasmodium falciparum infection in Tanzanian children. *Infect. Immun.* **74**, 3904 (2006).

[2] Sander A.F. *et al*. Multiple var2csa-Type PfEMP1 Genes Located at Different Chromosomal Loci Occur in Many Plasmodium falciparum Isolates. *PLoS One* **4**, e6667 (2009).

[3] Lavstsen T. *et al*. Plasmodium falciparum erythrocyte membrane protein 1 domain cassettes 8 and 13 are associated with severe malaria in children. *Proc. Natl. Acad. Sci. U. S. A.* **109**, E1791-800 (2012).
